# Bronchial artery aneurysm suggested to be caused by metalic tracheal stent migration

**DOI:** 10.1186/s40792-016-0247-1

**Published:** 2016-11-05

**Authors:** Kiyoshi Sato, Satoshi Fumimoto, Takehisa Fukada, Kaoru Ochi, Takayuki Kataoka, Yoshio Ichihashi, Hidetoshi Satomi, Takuya Morita, Nobuharu Hanaoka, Yoshikatsu Okada, Takahiro Katsumata

**Affiliations:** 1 Department of Thoracic Surgery, Osaka Medical College Hospital, 2-7 Daigaku-cho, Takatsuki, 569-8686 Japan; 2 Department of Thoracic Surgery, Yao Tokushukai General Hospital, Yao, Japan; 3 Department of Pathology, Osaka Medical College Hospital, Takatsuki, Japan; 4 Department of Surgery, Ujigawa Hospital, Uji, Japan

## Abstract

Occurrence of bronchial artery aneurysm is rare, and it has been detected in less than 1 % of all selective bronchial arteriography cases. Here, we present a case of a bronchial artery aneurysm caused by a tracheal stent migration. A 59-year-old man was operated on for esophageal cancer, where an esophageal-tracheal fistula occurred 1 week after operation. Surgical repair of the esophageal-tracheal fistula was performed using a muscle flap, but this not results in fistula closure. Consequently, a self-expanding covered metallic tracheal stent was implanted for rescue, and this resulted in fistula closure. After 1 year, there was frequent hemoptysis caused by migration of the stent. He was referred to our hospital where removal of the stent was planned. A sudden occurrence of massive bleeding from trachea occurred, and extracorporeal membrane oxygenation (ECMO) was used. Although removal of tracheal stent was performed successfully, the patient subsequently died from multi-organ failure. Post-mortem autopsy revealed that the massive bleeding is originated from the rupture of a bronchial artery aneurysm.

## Background

Self-expanding metallic tracheal stents have been used for benign or malignant tracheal stenosis and tracheal fistulas. Although the implantation of these stents are considered as a relatively safe treatment, severe complications such as bleeding and stent migration can sometimes occur. Here, we report a case of massive bleeding originating from the rupture of a bronchial artery aneurysm suggested to be caused by a metalic tracheal stent migration.

## Case presentation

**Fig. 1:**
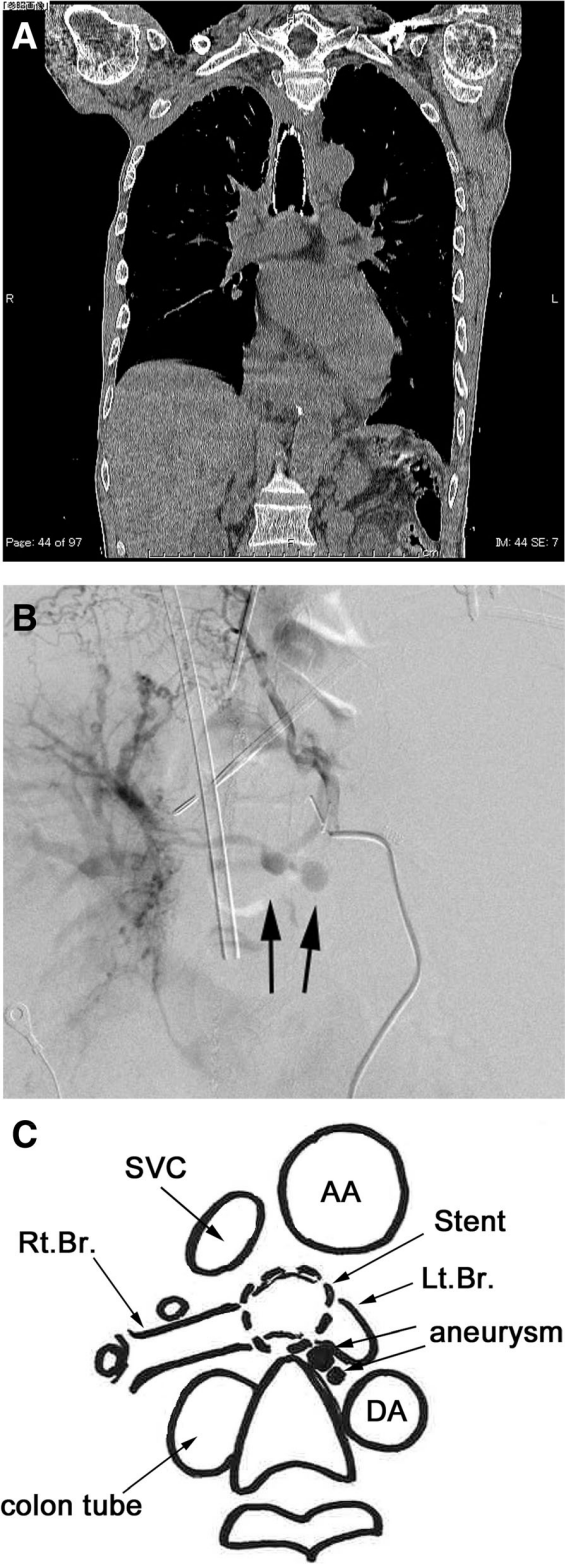
**a** A chest CT showing that the trachea was damaged by the distal side of the stent, which cut into the first carina of the trachea. **b** Bronchial arteriography revealed a bronchial artery aneurysm. **c** The schema shows bronchial artery aneurysms were located below the bronchial bifurcation and adjacent to the left main bronchus. *AA* ascending aorta, *DA* descending aorta

**Fig. 2:**
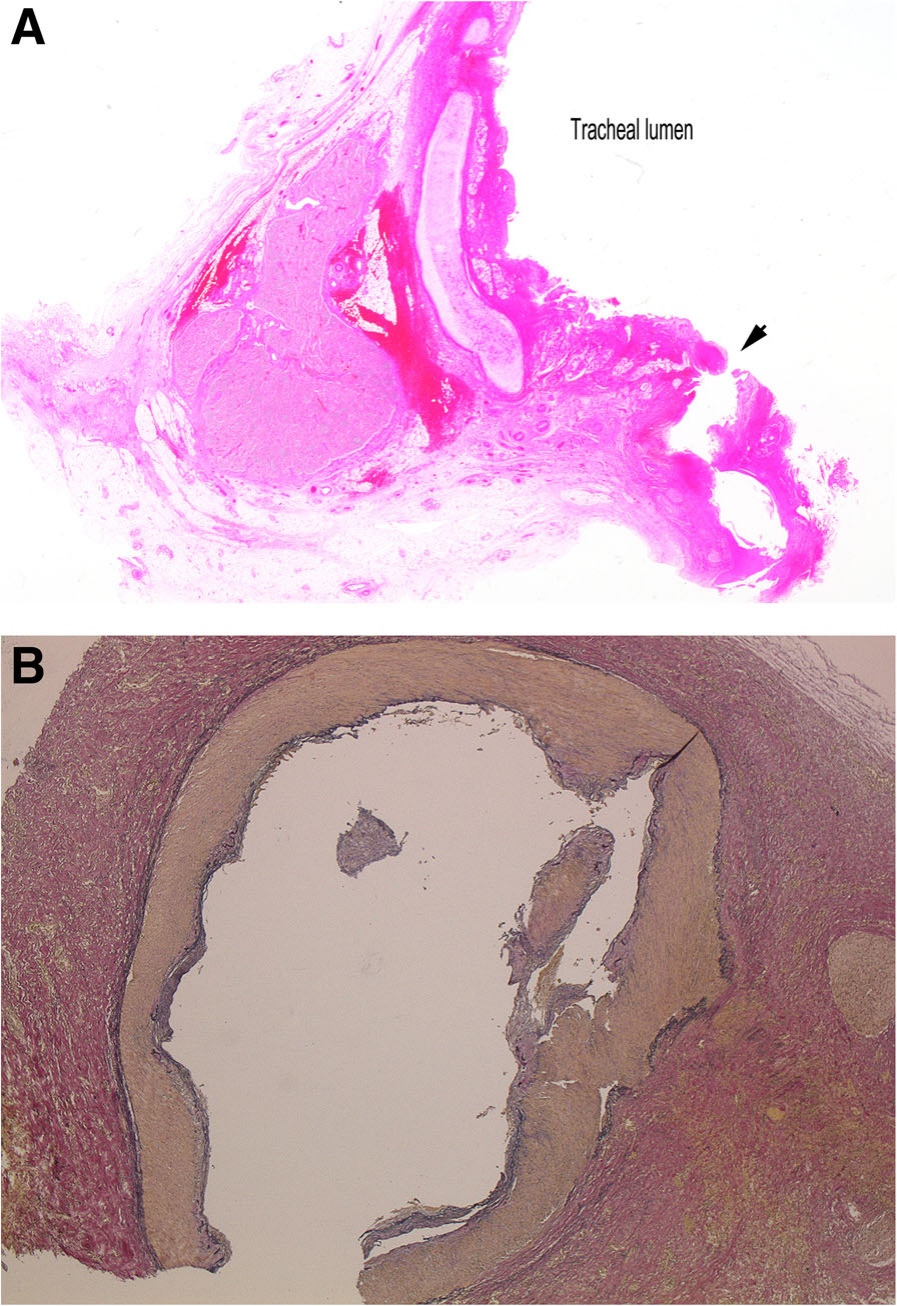
**a** Pathology findings of autopsy specimens showing meandering and expansion of the bronchial arterial wall and its perforation to the tracheal lumen (H&E staining, loupe image). **b** Immunohistochemical staining showing a decrease in connective tissue fibers which form an artery wall (EVG staining, ×20)

A 59-year-old man with squamous-cell esophageal carcinoma underwent thoracoscopic subtotal esophagectomy and posterior mediastinal reconstruction using a gastric tube in another hospital. Occurrence of a gastric tube-tracheal fistula was detected on postoperative day 7, and surgical repair of the esophageal-tracheal fistula was performed using the latissimus dorsi muscle flap, but fistula closure was not achieved. As there was progressive respiratory failure due to pneumonia, a self-expanding covered metallic tracheal stent (Ultraflex stent) was implanted for rescue. After recovery from pneumonia, another reconstruction procedure using the colon was performed, and the patient was discharged. One year later, there was frequent occurrence of hemoptysis, and CT scan showed that the trachea had been damaged by the distal side of the stent, which had cut into the first carina of the trachea (Fig. 1a). The patient was referred to our hospital and removal of the tracheal stent was planned. On the second day of hospitalization, a sudden massive hemoptysis occurred. As maintenance of oxygenation became difficult, emergency V-V ECMO was immediately introduced and removal of the tracheal stent was performed successfully. As tracheal bleeding from the bronchial artery was suspected, bronchial artery embolization (BAE) was performed, and a bronchial artery aneurysm was revealed by bronchial arteriography at the same time (Fig. 1b, c). Despite the performance of BAE, the bleeding could not be controlled and the patient died of multiple organ failure on the 15th hospital day. Post-mortem autopsy showed a bronchial artery aneurysm by histology and occurrence of aneurysm rupture into the bronchial lumen (Fig. 2a, b).

### Discussion

Recently, posterior mediastinal reconstruction is increasingly used as the reconstructive route for patients undergoing an esophagectomy for esophagus cancer. Occurrence of gastric tube-bronchial fistula after esophagectomy is a rare but potentially life-threatening complication that is specific to posterior mediastinal reconstruction, and incidence of this complication has been reported to be around 3 % [1]. This complication can result from perioperative tracheal injury, ischemic lesions due to devascularization of the trachea and main bronchus by extensive lymph node dissection, or leakage of the esophagointestinal anastomosis, and it may present early after esophagectomy or relatively late in the follow-up. Management strategy is dependent on the fistula site and size and the patient’s characteristics and general condition. Although various management approaches have been proposed, the optimal strategy remains controversial. Although some authors have reported successful cases of stent insertion for the fistula [2], appropriate surgical repair is required in many cases [3, 4]. Nonsurgical management, such as airway stents, can be considered for a small fistula with minimal clinical symptoms or patients who are unable to go through surgery. Silicone and self-expanding covered metallic stents have been used in patients with fistula after esophageal surgery. Insertion of a silicone stent such as a Dumon stent requires a rigid bronchoscope and specialized technical skills, and general anesthesia is usually required. In contrast, a self-expanding metallic stent is easy to insert through a delivery catheter using a flexible bronchoscope under local anesthesia. The most common complications of these stents are hemoptysis, stent migration, and stent granulation. In many reports, self-expanding metallic stents have a lower rate of migration and hemoptysis, but a higher rate of granulation as compared with silicone stents [5–7]. There are also several additional complications including stent fracture, trachea/broncho-esophageal fistula [8], broncho-vascular fistula [9], and difficulty in removal. Removal of metallic stents can also result in serious complications, including mucosal tears and severe bleeding. Consequent to these complications, the US Food and Drug Administration recommends that metallic stents should only be used in patients with benign airway disorders after thoroughly exploring all other treatment options.

In our patient, we initially suspected that the hemoptysis was caused by tracheal mucosa damage, but bronchial arteriography and autopsy revealed that the bleeding was caused by perforation of a bronchial aneurysm. Little is known about the cause of bronchial artery aneurysm; however, it is regarded to be associated with increased bronchial artery blood flow or weakening or injury of the vessel walls [10]. While congenital disease cannot be excluded in our case, weakening of the vessel walls by chronic irritation and inflammation from the stent is considered as the cause of the aneurysm. It is possible that the aneurysm might have been prevented if stent removal was performed early after the second reconstruction surgery. However, as previously mentioned, removal of a metallic stent is an inherently risky procedure, and therefore, stent removal is still subject to controversy.

## Conclusions

We present a case of bronchial artery aneurysm caused by tracheal stent migration. Bronchial artery aneurysm is rare but serious complication in patients with using metallic tracheal stent. The indication of the metallic tracheal stent insertion should be made sufficiently carefully for benign airway disorders such as our case.
